# Broad genomic workup including optical genome mapping uncovers a *DDX3X*: *MLLT10* gene fusion in acute myeloid leukemia

**DOI:** 10.3389/fonc.2022.959243

**Published:** 2022-09-09

**Authors:** Verena Nilius-Eliliwi, Marco Tembrink, Wanda Maria Gerding, Krzystof P. Lubieniecki, Joanna M. Lubieniecka, Stefanie Kankel, Thomas Liehr, Thomas Mika, Fotios Dimopoulos, Konstanze Döhner, Roland Schroers, Hoa Huu Phuc Nguyen, Deepak Ben Vangala

**Affiliations:** ^1^ Department of Medicine, Hematology and Oncology, Knappschaftskrankenhaus, Ruhr-University Bochum, Bochum, Germany; ^2^ Human Genetics, Ruhr-University Bochum, Bochum, Germany; ^3^ Jena University Hospital, Friedrich Schiller University, Institute of Human Genetics, Jena, Germany; ^4^ Department of Internal Medicine III, University Hospital Ulm, Ulm, Germany

**Keywords:** acute myeloid leukemia, optical genome mapping, *DDX3X*, *MLLT10*, FLT3-ITD, SUZ12, ARPP21, biphenotypic leukemia

## Abstract

In acute myeloid leukemia (AML), treatment decisions are currently made according to the risk classification of the European LeukemiaNet (ELN), which is based on genetic alterations. Recently, optical genome mapping (OGM) as a novel method proved to yield a genome-wide and detailed cytogenetic characterization at the time of diagnosis. A young female patient suffered from a rather unexpected aggressive disease course under FLT3 targeted therapy in combination with induction chemotherapy. By applying a “next-generation diagnostic workup“ strategy with OGM and whole-exome sequencing (WES), a *DDX3X: MLLT10* gene fusion could be detected, otherwise missed by routine diagnostics. Furthermore, several aspects of lineage ambiguity not shown by standard diagnostics were unraveled such as deletions of *SUZ12* and *ARPP21*, as well as T-cell receptor recombination. In summary, the detection of this particular gene fusion *DDX3X: MLLT10* in a female AML patient and the findings of lineage ambiguity are potential explanations for the aggressive course of disease. Our study demonstrates that OGM can yield novel clinically significant results, including additional information helpful in disease monitoring and disease biology.

## Introduction

Acute myeloid leukemia (AML) is a common hematologic malignancy with a mortality rate of approximately 50% (1, 2). Treatment has improved in recent years with the introduction of new drugs that target specific proteins altered by distinct genetic aberrations. This progress and the increasing knowledge of risk stratification urge for more accurate genetic testing at diagnosis and relapse. Treatment decisions are currently made in line with the risk classification of the European LeukemiaNet (ELN), which is based on disease-specific genetic alterations (3).

In a recent study, we compared standard cytogenetic methods with optical genome mapping (OGM) as a novel diagnostic method for structural genome analysis in AML and myelodysplastic syndrome (MDS) (4). Briefly, ultrahigh–molecular weight (UHMW) DNA is prepared and labeled genome-wide and sequence-specific with a fluorochrome dye. After being loaded on a chip and linearized through nanochannels by electrophoresis, the labeled DNA is captured by a fluorescence microscope and processed by different software algorithms against the human GRCh37/hg19 labeling pattern. Furthermore, different pipelines are used to detect structural variants (SVs) and copy number variants (CNVs). The coverage of this method is about 300×, and the mosaicism detection level currently is about 2% for SVs and 8% for CNVs, with a substantial number of CNVs also covered in the SV pipeline (4). Besides a substantial gain of relevant information by OGM compared to classical karyotyping, here, we detected a *DDX3X: MLLT10* gene fusion in a 21-year-old female AML patient. To date, description of *DDX3X: MLLT10* in AML is restricted to a few cases in male patients (5). Also, in acute lymphoblastic leukemia (ALL), where the fusion is more commonly involved, it is mainly found in male individuals (6, 7). To the best of our knowledge, this is the first time this aberration is described in a female AML patient. Strikingly, *DDX3X* is a gene that escapes X-inactivation in women (8). Accordingly, Brandimarte et al. (6) hypothesized that *DDX3X* translocations might be a leukemic driver due to the lack of a second gene copy in male individuals (6, 9). Apart from that, both genes, *DDX3X* and *MLLT10*, separately have been observed to play a role in a whole range of different hematologic and solid malignancies (10–12).

Based on cytomorphology, immunophenotypic analysis, and histopathology, the disease in our patient was classified as CD33+, CD34+, CD38+, CD117+ AML with aberrant CD7 expression. By OGM, different genetic alterations related to T-lineage commitment were unraveled. Interestingly, the disease course appeared to be worse than expected. To improve our understanding of the aberrations and their contribution to leukemogenesis, we further analyzed the bone marrow sample by fluorescence *in situ* hybridization (FISH), whole-exome sequencing (WES), and consecutive OGM analysis. The purpose of this report is to provide insights into the benefits of a “next-generation diagnostic workup“ for hematologic diseases using two different genome-wide high-resolution technologies and to find out whether its application might result in additional information useful for clinical decision-making.

## Methods

Genetic standard diagnostics, such as karyotyping and PCR panels, are presented in the *Results* section and were performed at the University Hospital Knappschaftskrankenhaus Bochum and in collaborating laboratories. The patient gave written informed consent for genetic diagnostics and publication of results.

### Optical genome mapping

OGM was performed as part of a recently published study at the initial time point (first time point) of AML diagnosis with bone marrow aspirate (BMA) and analyzed using Bionano Access v1.6. After the end of leukocyte nadir following reinduction treatment, consecutive OGM analysis of BMA was performed (second time point). UHMW DNA was isolated using an isolation kit from Bionano Genomics following the manufacturer’s protocol. The DNA motif CTTAAG was labeled fluorescently by direct label and stain technology using a pattern recognition enzyme according to the manufacturer’s instructions [direct labeling enzyme 1 (DLE-1), Bionano Genomics]. Optical scans were performed on a Saphyr platform (Bionano Genomics) using a G2.3 chip. Raw molecule data generated by OGM from samples collected at two different time points were computed using the Rare Variant pipeline (RVP) of Bionano Solve v3.7 and analyzed by the latest Bionano Access release v1.7, adding allele frequency estimates for SVs and an optimized nomenclature. OGM data showed an effective post-analysis 403-fold coverage at the initial time point and a 395-fold coverage after induction therapy. Quality metrics were reached for both samples. GRCh37/hg19 human genome reference was used for alignment.

Output SVs and CNVs were filtered against masks for known regions of high variance and default confidence cutoffs in Bionano Access v1.7 (insertion 0, deletion 0, inversion 0.7, duplication -1, intra-fusion 0.05, inter-translocation 0.05). SVs were filtered for absence in a control population consisting of 179 healthy controls provided by the manufacturer (Bionano Genomics). The remaining SVs were evaluated for their confidence, uniqueness, gene annotation overlaps, and presence in the Database of Genomic Variants (DGV). T-cell receptor (TCR) loci SVs were reported without filtering against controls, as somatic mutations are part of physiological T-cell maturation processes. All CNVs after CNV mask filtering using default confidence cutoffs (0.5 Mbp for size and 0.99 for confidence) were reported.

### Fluorescence *in situ* hybridization

To confirm the gene fusion *DDX3X: MLLT10*, FISH analysis on interphase nuclei was performed. FISH probes were hybridized to bone marrow smears according to manufacturer’s instructions (MetaSystems, Altlussheim, Germany). A 193-kilobase probe covering the whole *DDX3X* locus including STS markers HUMSWX1304/RH1146 to DBX was synthesized (DDX3X-20-AQ; Empire Genomics), and a commercially available probe for the detection of *KMT2A::MLLT10* [XL t (10;11) MLLT10/KMT2A DF, MetaSystems, Altlussheim, Germany] was applied.

### Whole-exome sequencing

A WES library was constructed using SureSelectXT Human All Exon V7 (Agilent Technologies) and sequenced on NovaSeq 6000 instrument (Illumina). Sequence alignment, variant calling, and annotation were performed using Varvis bioinformatics pipeline v1.20.0 (Limbus Medical Technologies). For evaluation of myeloid cancer-related mutations, the coding sequences of 54 genes based on a commercially available myeloid NGS panel (TruSight Myeloid Sequencing Panel, Ilumina) and four case-specific genes based on OGM results (*DDX3X*, *MLLT10*, *NF1*, *SUZ12*) were analyzed for SNVs and CNVs. All detected SNVs with a population frequency <1% in gnomAD database were extracted and rated according to current cancer sequence variant interpretation standards of AMP consensus for myeloid cancer-related genes. *DDX3X:MLLT10* breakpoint search was done using the Varvis alignment by manual inspection in Integrative Genome Viewer (IGV), and fusion was confirmed using BLAT search tool University of California Santa Cruz (UCSC) and Sanger Sequencing (3500xL Genetic Analyzer, Applied Biosystems) (detailed information in **Supplementary Material 1**) (13, 14).

### T-cell receptor clonality and junction analysis

To confirm clonal TCR TRD V(D)J recombination, Sanger sequencing was performed using primers for the amplification of *TRDV1* and *TRDJ1* generated using sequences of a EuroClonality-NGS validation study (sequences available upon request) (15). Sequencing was performed on a 3500xL Genetic Analyzer (Applied Biosystems). Sequence was analyzed using IMGT/V-Quest v3.5.29 incorporating TCR mutation junction analysis and reading frame evaluation (16).

## Results

### Patient characteristics and disease course

**Figure 1:**
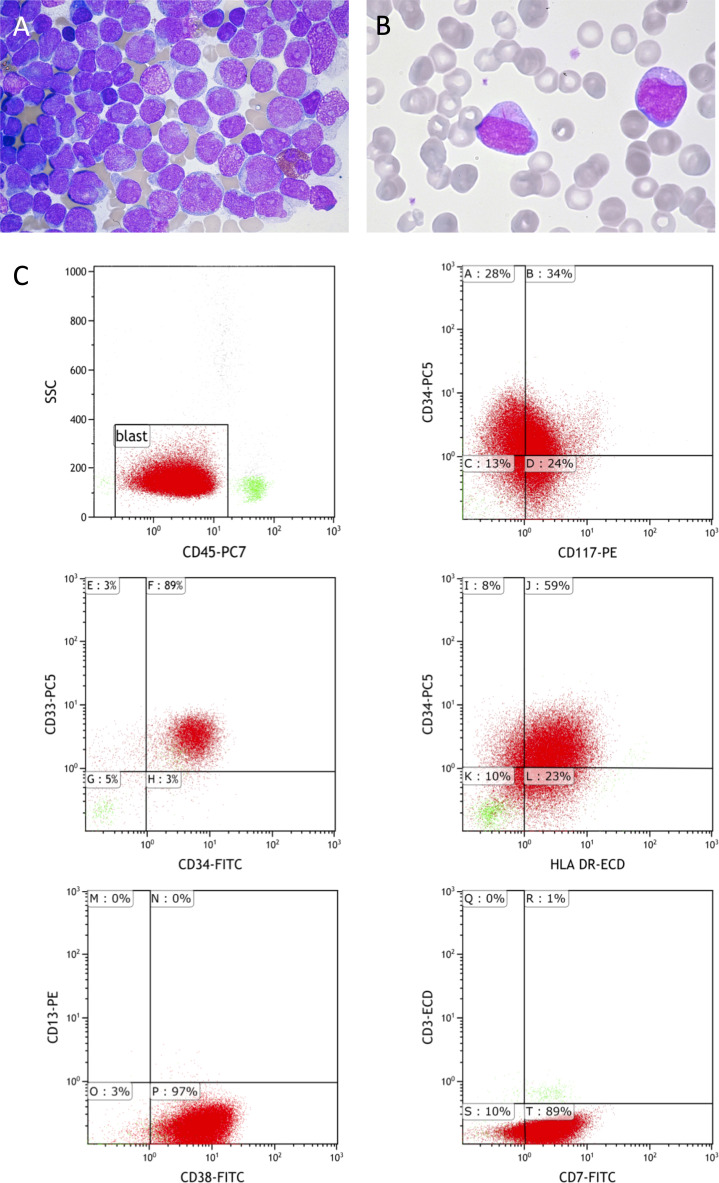
Cytology and immunophenotyping at initial diagnosis. Cytology of bone marrow **(A)** and peripheral blood **(B)** at initial diagnosis showing a subtotal infiltration of myelomonocytic blasts **(A)** and a blast with an Auer rod **(B)**. Immunophenotyping of bone marrow aspirate by flow cytometry **(C)** showing the gating strategy and relevant markers leading to the diagnosis of AML. ECD, extracellular domaine; SSC, Side scatter; PE, Phycoerythrine; FITC, Fluoresceinisothiocyanate.

At initial diagnosis (first time point) in December 2020, the 21-year-old female patient presented with peripheral leukocytosis of 81 × 10^9^/L accompanied by thrombocytopenia concomitant to a bone marrow infiltration of >90% with myelomonocytic blasts including some Auer rods (**Figures 1A, B** **)**. Immunohistological workup revealed a weak expression of lysozyme and myeloperoxidase. Flow cytometry showed the expression of CD33, CD34, CD38, HLA-DR, and CD117, as well as aberrant expression of CD7 (**Figure 1C**). CD1a, surface and cytosolic CD3, and TCR were not detected. Cytogenetics reported a normal karyotype in the bone marrow sample, but only three metaphases could be evaluated. Molecular fragment length analysis subsequent to PCR amplification showed *FLT3*-ITD^high^ (ratio 0.759) and biallelic heterozygous *CEBPA* mutations. Fusion genes were not detected in a standard RT-PCR panel (Menotype AMLplexQS) and neither *IDH1*, *IDH2*, *NPM1*, *FLT3*-TKD (PCR/fragment length analysis) nor *ASXL1*, *RUNX1*, *TP53* [next-generation sequencing (NGS)] mutations were reported.

Based on these initial diagnostic results, the patient was grouped as high risk according to ELN 2017 risk classification and included in a clinical trial receiving gilteritinib in addition to the standard “3 + 7” induction therapy (daunorubicin 180 mg/m^2^ and cytarabine 1,400 mg/m^2^). Although not reaching a blast clearance after initial induction, the patient was allowed to stay on study and was reinduced with daunorubicin (180 mg/m^2^) and intermediate-dose cytarabine (12 g/m^2^). However, despite combining intensive chemotherapy and targeted *FLT3*-ITD treatment with a Tyrosine Kinase Inhibitor (TKI), complete remission could not be achieved by this approach and the patient showed a persistence of blasts of about 20% (second time point) similar immunophenotypic expression profile (**Supplementary Figure S1**). Thus, salvage therapy according to FLAG-Eto-protocol (fludarabine 150 mg/m^2^, cytarabine 10 g/m^2^, etoposide 300 mg/m^2^, granulocyte colony stimulating factor (G-CSF) starting day 10) was initiated. Fortunately, complete remission was reached, and after identification of an human leukocyte antigen (HLA)-matched unrelated donor, the patient proceeded to allogeneic hematopoietic stem cell transplantation (allo-HSCT) following conditioning treatment including fludarabine (150 mg/m^2^) and treosulfan (36 g/m^2^). Graft versus Host Disease (GvHD) prophylaxis consisted of antithymocyte globulin (ATG), methotrexate (MTX), and ciclosporin A. After recovering without any major complications, maintenance treatment with sorafenib was started.

Interestingly, the patient developed hyperglycemia and was diagnosed with maturity-onset diabetes of the young (MODY) type 3 that correlated with the detection of a germline *HNF1A* variant (NM_000545.8 c.457C>T), previously rated as pathogenic using gene American College of Medical Genetics and Genomics (ACMG) criteria (17). Currently, 400 days after allo-HSCT, the patient is weaned off immunosuppressants, has recovered fully, and remains molecularly disease-free. Patient characteristics are summarized in **Table 1**.

**Table 1:** Clinical characteristics.

	First time point initial diagnosis	Second time point after reinduction therapy
Leukocyte count/L	81 × 10^9^	5 × 10^9^
Hb mmol/l	8.14	6.09
Thrombocyte count/µl	99,000	205,000
Histopathology	>90% blasts	30% blasts
Cytology	>90% blasts, single Auer rods	20% blasts, no Auer rods
Flow cytometry	>90% blasts: CD33, CD34, CD38, HLA-DR, CD117, CD7 pos. CD1a, CD3, cyCD3 neg.	12% blasts: CD33, CD34, CD38, HLA-DR, CD117 pos. CD1a, CD3, cyCD3, CD7 neg.
Karyotyping	46,XX [3]	46,XX [27]
*FLT3*-ITD	0.76 *FLT3*-ITD^high^	0.05 *FLT3*-ITD^low^
RT-PCR panel/Fragment length analysis	*CEBPA* mut. biallelic, heterozygous	*CEBPA* mut. biallelic, heterozygous

Most important findings by standard diagnostics of both time points. RT-PCR panel covering the following aberrations: *FLT3-*TKD*, NPM1*, *CBFB-MYH11*, *RUNX1-RUNX1T1*, *PML-RARA*, *CEBPA*, *IDH1*, *IDH2*, *BCR-ABL*, *KMT2A-MLLT3*. Hb, hemoglobin; ITD, internal tandem duplication.

### Optical genome mapping

In consecutive analyses, 23 and 28 SVs that were absent in healthy control samples were identified in our patient. After sorting out SVs commonly present in the DGV, SVs with low confidence, duplicate SVs, and SVs not overlapping annotated genes, eight SVs remained. All of them were detected at both diagnostic time points. Variant allele frequencies (VAFs) of the SVs of interest ranged from 42% to 53% (first time point) and from 8% to 16% (second time point), corresponding to heterozygous presence in blasts; the histologically estimated blast proportions were given as >90% and 20%–30%, respectively.

**Figure 2:**
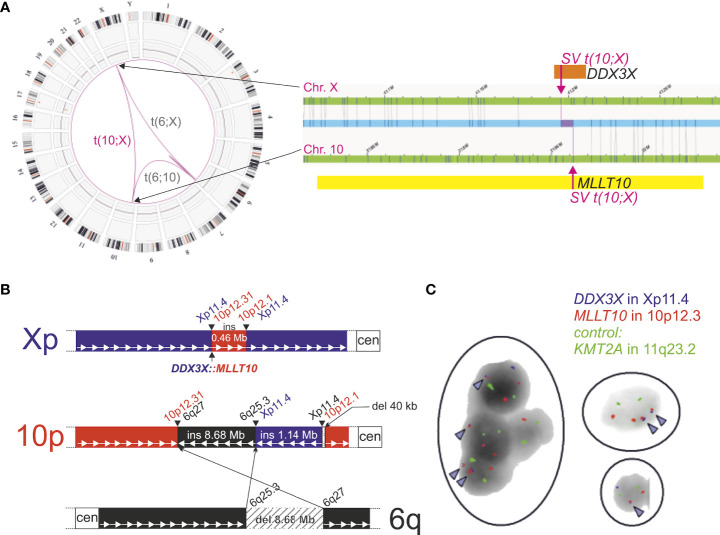
Detection of *DDX3X: MLLT10* gene fusion by optical genome mapping at the first time point and confirmation by FISH analysis. **(A)** Circos Plot (left) and detailed chromosome view SV visualization (right) of *DDX3X: MLLT10* in Bionano Access v1.7 (Translocation Confidence: inter: 0.65, intra: 0.3) filtered for absence in controls. **(B)** Schematic overview of structural rearrangement. A 0.46-Mbp fragment of Chr. 10p including *MLLT10* is transferred to Chr. Xp, fusing it to *DDX3X*. Furthermore, on Chr. 10p, an 8.68-Mbp fragment of Chr. 6q and a 1.14-Mbp fragment of Xp are inserted. Next to this insertion, a 40-kbp fragment is deleted. Both insertions are inverted and not in frame. **(C)** Interphase FISH analysis on bone marrow smear demonstrates *DDX3X: MLLT10* gene fusion. Red signals (corresponding to *MLLT10* at 10p12.13) and blue signals (corresponding to *DDX3X* at Xp11.4) are visualized adjacently and are indicated by arrowheads. In addition, green *KMT2A* control signals are not located in proximity to red *MLLT10* hybridization signals, indicating the lack of involvement of *KMT2A* in the structural rearrangement. Analyzed cell nuclei reveal heterogeneous hybridization signals due to subclonal events.

A balanced rearrangement overlapping *MLLT10* and *DDX3X* was represented in OGM by five interchromosomal-translocation SVs and one intrachromosomal-translocation SV with VAFs between 45% and 53%. As revealed by OGM, a complex rearrangement involving three chromosomes was found, involving chromosomes X, 10, and 6, resulting in a *5’*-*DDX3X: MLLT10-3’* gene fusion on X-chromosome visualized in **Figures 2A, B**. Segments of different sizes are exchanged between chromosome Xp (1.14 Mbp) and 10p (0.46 Mbp). Additionally, a deleted piece of 8.68 Mbp of chromosome 6q material was found to be inserted into the derivative chromosome 10p.

Furthermore, a 1.4-Mbp deletion including the Cosmic Cancer Gene Census hallmark genes *NF1* and *SUZ12* was detected at both time points of OGM analysis (18). These SVs were also confirmed by corresponding copy number losses (manually detectable at the first and second time points but only called above the confidence threshold at the first time point due to the lower allele fraction at the second time point). Moreover, an 84-kbp SV deletion including the 5’ part of *ARPP21* was detected in 42% and 8% allelic fraction at the first time point and second time point, respectively. The 3’ region containing one of two different genomic loci for micro-RNA 128 (miRNA128-2) and a polymerase III intronic promoter region allowing for *ARPP21*-independent transcription were preserved (19, 20).

SVs of the TCR regions *TRA(D)*, *TRG*, and *TRB* were analyzed without filtering against controls. At *TRA(D)* locus, two different SV deletions were identified at both time points with corresponding VAF proportions compared to histologically determined blast percentages. One of these SVs seemed to represent a somatic *TRDV1-TRDJ1* V(D)J recombination. The TRG locus contained one matching SV deletion with VAFs of 96% and 49% at both time points, respectively, which were likely overestimated by the software (196 and 16 molecules representing SV). Insertion SVs found at the TRB locus were less meaningful (5 and 3 insertion SV calls).

Sanger sequencing confirmed the suspected *TRDV1-TRDJ1* recombination. Sequence analysis using IMGT/V-Quest-tool called an out-of-frame/nonsense mutation and junction analysis suspected *TRDD2* and *TRDD3* involvement.

### Fluorescence *in situ* hybridization

In order to confirm OGM results regarding the *DDX3X: MLLT10* gene fusion, FISH analysis was performed on interphase nuclei for the visualization of *MLLT10* and *DDX3X* loci (**Figure 2C**). Additionally, a probe for *KMT2A* was co-hybridized to examine a potential rearrangement involving *MLLT10’s* most common fusion partner *KMT2A*. These results confirmed the *DDX3X: MLLT10* gene fusion without evidence for involvement of the *KMT2A* gene in this rearrangement.

### Whole-exome sequencing

After filtering for coding mutations, sequencing errors, and occurrence in <1% of gnomAD, seven SNVs and one CNV remained after analysis of the Cancer Virtual Panel.

One N-terminal frameshift mutation in *CEBPA* was called by Varvis software [c.308dup; p.Gly104fs, 35.3% allele frequency (AF)]. Another N-terminal frameshift mutation in *CEBPA* was not called (minimum called AF 7% at high coverage) but visible in approximately 7% of reads of the alignment visualized in IGV (c.116_117delinsA; p.Pro39fs) (14).

The *FLT3*-ITD was called with an AF of 57.7%. A 1.37-Mbp spanning loss including *NF1* and *SUZ12* was also detected by Varvis software (CN 1, no mosaic). In the remaining copy of *NF1*, no mutations were detected. On the other hand, in *SUZ12*, a c.682C>T (p.Pro228Ser) missense mutation at AF of 89.7% was detected (tier 3 VUS), which is absent in gnomAD. This result corresponded to the CNV loss in >90% blast material.

Furthermore, an *MLLT10*:c.2665G>A p.Val889Met missense mutation in exon 21 (tier 3 VUS) was observed, which is extremely rare in gnomAD. Also, the *DDX3X: MLLT10* genomic breakpoint in exon 4 (*DDX3X*) and intron 10 (*MLLT10*) was manually visible in the alignment and proven by Sanger sequencing.

Combining those genetic diagnostic methods led to the finding of several additional aberrations. *DDX3X: MLLT10* gene fusion and deletions in *NF1*, *SUZ12*, and *ARPP21* were detected with OGM and confirmed by WES. The gene fusion was additionally seen with FISH. Clonal TRD recombination as detected by OGM was validated using Sanger sequencing. The *FLT3*-ITD and biallelic *CEBPA* mutation found by standard RT-PCR were confirmed by WES.

## Discussion

Acute leukemias have undergone tremendous changes regarding diagnostics and to a lesser extent treatments over the last years. We have recently described OGM as a potential tool to detect SVs on a genome-wide level with sufficient depth and high concordance to currently used methods as classical karyotyping (4). By implementing OGM in our diagnostic algorithm in combination with WES as part of an exemplary next-generation diagnostic workup, we could detect a *DDX3X: MLLT10* gene fusion, to the best of our knowledge, for the first time in a female AML patient. Both genes, *DDX3X* and *MLLT10*, have been previously described to be involved in hematological and non-hematological neoplasia (10, 11).

In detail, *DDX3X* has previously been described as a regulator of RNA metabolism (21). Its role as a tumor suppressor and simultaneously as an oncogenic driver is complex and yet to be fully understood (10). The helicase interacts with important key pathways including p53 and kirsten rat sarcoma virus homologue (KRAS) (22–25). Somatic mutations were found in a variety of cancers including medulloblastoma and hematological neoplasia such as acute and chronic lymphocytic leukemia (26, 27). *DDX3X* appears to be epigenetically silenced in renal cell carcinoma and has been discussed as a potential therapeutic target (28, 29).

Rearrangements involving the *MLLT10* gene are recurrent in ALL (more common in T-lineage ALL) and less often in AML (typically pediatric) (12, 30, 31). Regardless of the partner gene, *MLLT10* rearrangements appear to be associated with an adverse outcome in AML (5). MLLT10 is involved in histone modification by regulating the function of the histone methyltransferase DOT1L, the only methyltransferase known to methylate H3K79. In *MLLT10*-rearranged leukemia, DOT1L induces the transcription of genes involved in cell cycle progression (especially genes in the Homeobox A Cluster) (32–34). This might in the future have clinical implications, as a recent phase I trial with pinometostat, as a specific inhibitor of DOT1L, showed some promising results (35).

**Figure 3:**
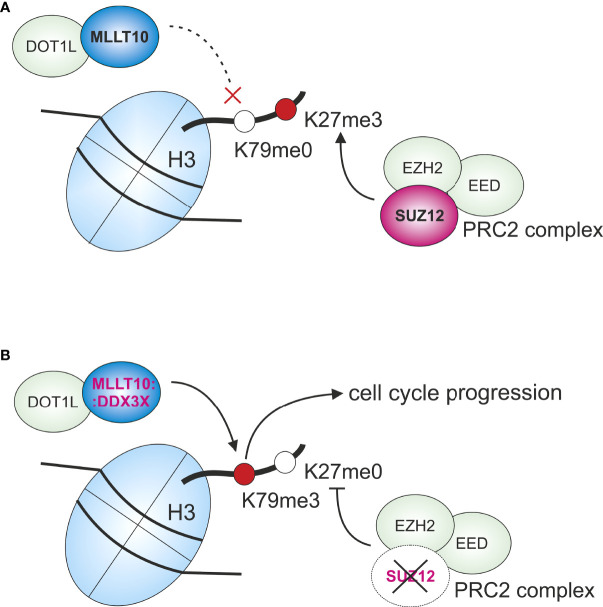
Graphic overview of interactions regarding histone 3 methylation with the participation of SUZ12 and MLLT10 under physiological conditions **(A)** and in the leukemic cells as suspected here **(B)**. **(A)** Under physiological conditions, PRC2 complex with SUZ12 and EED, and EZH2 methylates H3K27. This methylation abrogates the binding of DOT1L complex. **(B)** With SUZ12 being deleted, H3K27 is not methylated, which leads to binding of DOT1L complex and thereby to methylation of H3K79. This binding is probably additionally intensified because of *DDX3X: MLLT10* gene fusion, enabling transcription of genes that promote, among other effects, cell cycle progression, e.g., *HOXA* genes. H, histone; Me, methylated.

As in most ALL cases, reports of *DDX3X: MLLT10* fusions in AML are restricted to a few cases in male patients (5, 6). Apart from the *DDX3X: MLLT10* gene fusion, OGM could also unravel deletions in *ARPP21*, *NF1*, and *SUZ12*. SUZ12 is part of the polycomb repressive complex 2 (PRC2) that in turn is responsible for the methylation of H3K27 (36, 37). This methylation enables DOT1L with its complex to bind and exert its function as a transcription initiator for H3K79 (34). Thus, the *SUZ12* deletion and the *DDX3X: MLLT10* gene fusion might act synergistically in histone modification and could explain the aggressive course of disease (**Figure 3**).

ARPP21 is known to positively regulate *PHF6*-mRNA. It has a physiological counterpart, miRNA128, which is among others located at the 3′ end of *ARPP21* (20). OGM here clearly shows that the deletion does not affect miRNA128 and its intronic polymerase III-dependent open reading frame (19). Altogether, this might lead to a downregulation of *PHF6*-mRNA, which is a known tumor suppressor (38, 39).

Intriguingly, most of the findings uncovered by our next-generation diagnostic workup have rarely been associated with AML but are more common in early T-lineage ALL. In pediatric T-lineage ALL, *DDX3X* is the second most common fusion partner for *MLLT10* after *PICALM* (40). Furthermore, *SUZ12* and *PHF6* have been described in the context of T-ALL, with *PHF6* being one of the most frequently mutated or deleted genes in T-lymphoblastic leukemia and less often in AML and other myeloid neoplasia (38, 41, 42). Furthermore, OGM showed deletion SVs in the TCR regions representing clonal V(D)J recombinations. Clonal recombination of TRD and TRB could be indicative of an early stage in T-cell development. Combined with the immunophenotypic findings of CD34 and CD38 as well as lack of CD1a expression, these findings support the hypothesis that the leukemic blasts are in a pro-thymocyte (DN2) state (43, 44).

Lineage attribution today is mostly based on immunophenotyping, and AML and ALL are further classified according to genetic alterations relevant for further prognosis. This in turn leads to treatment protocols that 1) are lineage-specific, 2) are more or less intensive according to the genetic risk stratification, and 3) might include targeted therapies if actionable variants are detected. In the case described here, despite an aberrant CD7 expression in flow cytometry, routine diagnostics including cytology (Auer rods), immunohistology (myeloperoxidase and lysozyme expression), immunophenotyping by flow cytometry (**Figure 1**, **Supplementary Figure S1**), and the mutational profile (*FLT3-*ITD, *CEBPA*) led to the diagnosis of an AML. This is in line with the WHO 2016 classification, and even the older criteria of the European Group for the Immunological Classification of Leukemias (EGIL) would not have diagnosed a mixed-phenotype acute leukemia (MPAL) but an AML with aberrant coexpression of lymphatic markers due to the CD7 expression (45, 46). However, although being by far more prevalent in AML, neither Auer rods nor *FLT3*-ITD or *CEBPA* variants are exclusive to myeloid differentiation but have been previously described in ALL, especially in early T-cell precursor (ETP)-ALL (47–50).

In clinical practice, commitment to a diagnosis is necessary for defining the optimal treatment. Despite the arguably best available treatment protocol by adding FLT3-targeted therapy to standard “7 + 3” induction, treatment response was unsatisfying, without sufficient blast clearance. However, FLAG-containing regimens with consolidating allogeneic transplantation and sorafenib maintenance treatment in case of activating *FLT3* mutations not only are regular salvage approaches in refractory AML but also have been described as a successful therapy in ETP-ALL (51).

**Figure 4:**
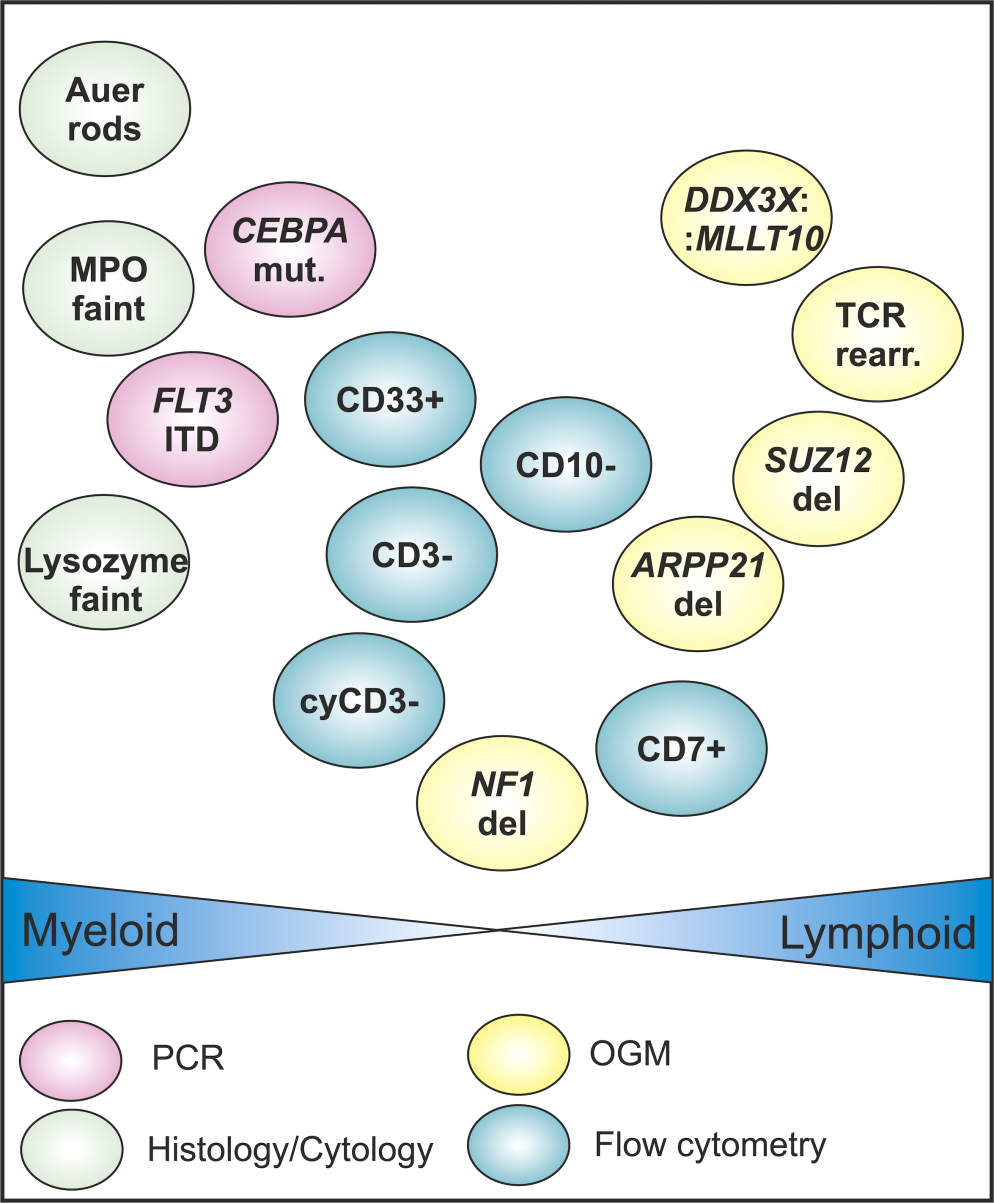
Graphical overview of selected diagnostic results obtained by different methodologies: results by optical genome mapping (OGM) are stained in yellow, PCR panel results in pink, histology results in green, and flow cytometry results in blue. The findings are positioned depending on their probability of occurrence in myeloid and lymphoid cells. The positioning reflects a general orientation and is not scaled. OGM findings are rather found on the lymphoid side. Cy, cytoplasmic; del, deletion; ITD, internal tandem duplication; MPO, myeloperoxidase.

The immunophenotypic profile (including immunohistochemistry and flow cytometry) in this case was indicative of a myeloid differentiation (**Figure 1C**, **Figure 4**). Treatment with FLAG-Eto as salvage therefore was a straightforward approach. Compared to that, the initial diagnosis of a T-lineage disease not only would have led to a different treatment protocol but also might have changed the approach in the treatment-refractory situation with substances such as nelarabine. Thus, our here proposed broad genomic workup utilizing whole-genome cytogenetics and WES might in the future lead to a more distinct classification of disease and might help to overcome the flaws of immunophenotyping in certain situations. The detection of genetic changes typical for myeloid and early lymphoid differentiation as presented here could hint to a stem cell-like myeloid-lymphoid precursor, as has been very recently proposed by Genescà and Starza (52). This in turn could lead to more personalized approaches in treatment, omitting unwanted toxicities and improving the outcome for the patient (53).

Without OGM as a whole genome-based method with high resolution and deep coverage, the *DDX3X: MLLT10* gene fusion and other genetic aberrations not covered by routine diagnostics would have been missed. In this work, FISH, WES, and Sanger sequencing were applied as conformational diagnostics. Notably, the FISH probes used are far from any routine use. Thus, not only does OGM have the potential to improve the detection of novel aberrations but also it renders multiple other methods somewhat uncalled for.

In summary, by implementing OGM as a cytogenomic method, we detected SVs in genes involved in histone modification (*DDX3X: MLLT10*, *SUZ12)*, offering a functional explanation for aggressiveness of disease in this case. Furthermore, these and other detected variants (*ARPP21*, TCR variants) in combination with mutated myeloid genes also typical for ETP-ALL clearly hint lineage ambiguity, which was missed by routine diagnostic procedures relying on immunophenotyping for lineage discrimination. In conclusion, a next-generation diagnostic approach as proposed here has the potential to not only detect novel genetic variants but also improve risk stratification and thus individualized treatment.

## Data availability statement

The data presented in the study are deposited under http://zenodo.org under accession number 6421158 387.

## Ethics statement

This study was reviewed and approved by Ethik-Kommission der Medizinischen Fakultät der Ruhr-Universität Bochum Gesundheitscampus 33. The patients/participants provided their written informed consent to participate in this study. Written informed consent was obtained from the individual(s) for the publication of any potentially identifiable images or data included in this article.

## Author contributions

VN-E, MT, WG and DBV prepared the manuscript. FD, TM, VN-E, DBV, RS and KD collected patient data. KL, MT and JL analyzed WES data. TL and SK performed and analyzed FISH experiments. HN, RS and DBV contributed to conception and design of the study. All authors contributed to the article and approved the submitted version.

## Funding

We thank FORUM of the Faculty of Medicine at the Ruhr-University Bochum for funding MT (medical doctoral thesis program) and VN-E as part of the Female Clinician Scientist program.

## Acknowledgments

Thanks to Iris Over for expert technical assistance and the patient for participating in the study.

## Conflict of interest

DV received speaker’s honoraria from Roche, consultant’s honoraria from Pfizer, Bristol Myers Squibb and Gilead as well as travel support and congress registration fees from Gilead and Celgene.

The remaining authors declare that the research was conducted in the absence of any commercial or financial relationships that could be construed as a potential conflict of interest.

## Publisher’s note

All claims expressed in this article are solely those of the authors and do not necessarily represent those of their affiliated organizations, or those of the publisher, the editors and the reviewers. Any product that may be evaluated in this article, or claim that may be made by its manufacturer, is not guaranteed or endorsed by the publisher.

## Supplementary material

Supplementary Figure 1 Immunophenotyping of bone marrow aspirate from the second time point before salvage chemotherapy. Despite a visible neutrophile-cloud compared to initial diagnosis, the blast-population with a similar expression profile is still clearly visible.
