# Supplementary figures and images for: Broad genomic workup including optical genome mapping uncovers a DDX3X: MLLT10 gene fusion in acute myeloid leukemia
Source: Front Oncol. 2022 Sep 9;12:959243. doi: 10.3389/fonc.2022.959243 (PMC9501710; doi:10.3389/fonc.2022.959243)

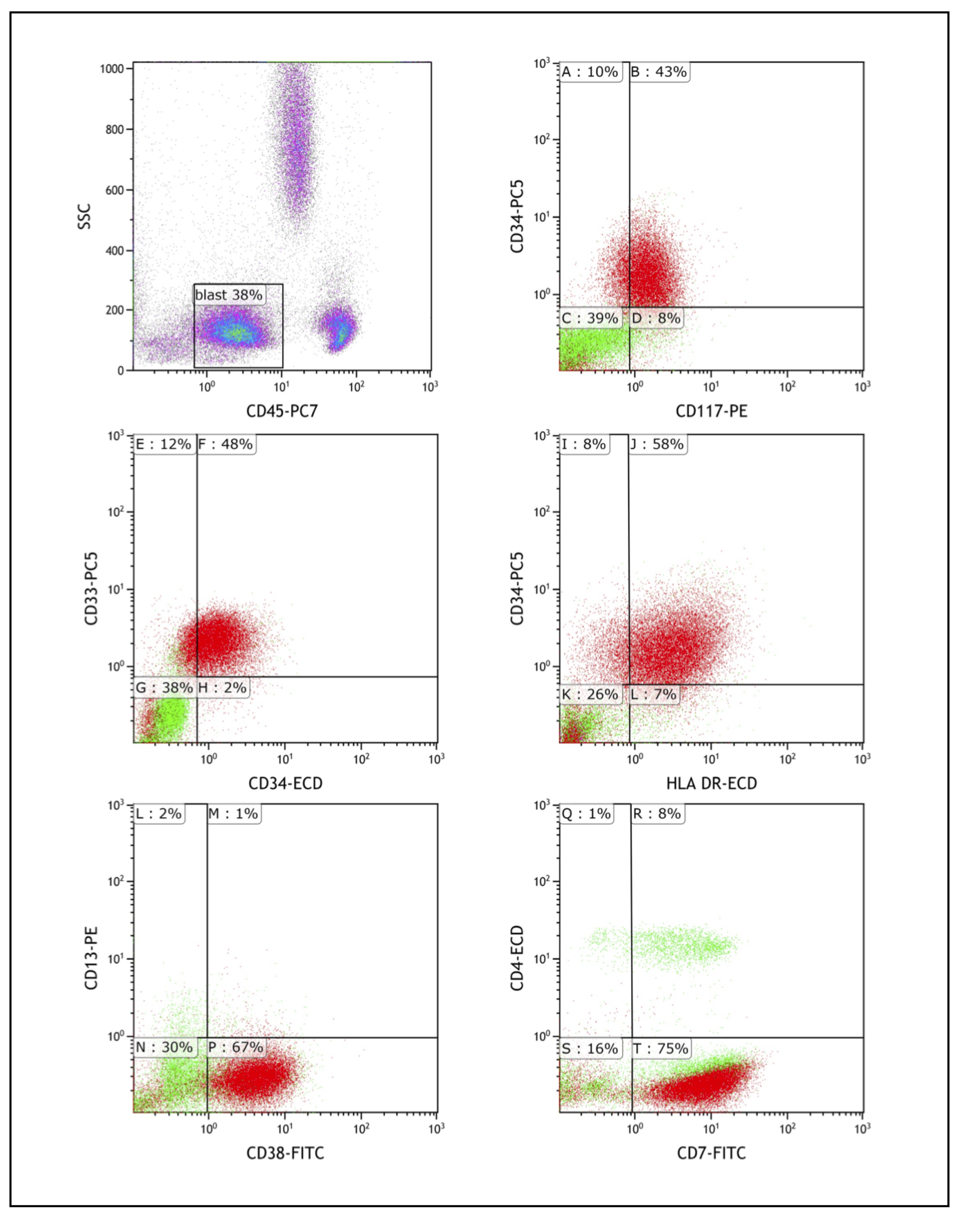

Supplement: Supplementary Figure 1 — Immunophenotyping of bone marrow aspirate from the second time point before salvage chemotherapy. Despite a visible neutrophile-cloud compared to initial diagnosis, the blast-population with a similar expression profile is still clearly visible. [file Image_1.jpeg]
